# Amelanotic Nodular Melanoma Over the Knee Region: A Case Report of a Diagnostic Challenge From the United Arab Emirates

**DOI:** 10.7759/cureus.85630

**Published:** 2025-06-09

**Authors:** Khadiga Abdelmonem, Loay Ghalyoun, Divyashri Ramanathan Nagarajan, Sonia Otsmane, Joaquin Picazo-Yeste

**Affiliations:** 1 Medicine, Burjeel Medical CIty, Abu Dhabi, ARE; 2 Medicine, Burjeel Medical City, Abu Dhabi, ARE; 3 Medical Oncology, Burjeel Medical City, Abu Dhabi, ARE; 4 General Surgery, Burjeel Medical City, Abu Dhabi, ARE

**Keywords:** knee lesion, misdiagnosis, nodular melanoma, s: amelanotic melanoma, sentinel lymph node, united arab emirates

## Abstract

Amelanotic melanoma (AM) is a rare and aggressive subtype of cutaneous melanoma that lacks the characteristic pigmentation, which makes it challenging to diagnose, often resulting in delayed treatment. Nodular melanoma (NM), a subtype of AM, is associated with a poorer prognosis and is infrequently reported in the Middle East and North Africa (MENA) region. AM is a rare and aggressive subtype of cutaneous melanoma, accounting for approximately 1-8% of all melanoma cases. We present the case of a 39-year-old Filipino female with a slowly enlarging, skin-colored lesion on her right patella, initially misdiagnosed as a dermatofibroma. The lesion was later confirmed as type IIIb cutaneous amelanotic nodular melanoma via histopathology and immunohistochemistry, with a Breslow thickness of 5 mm and Clark level IV invasion. Sentinel lymph node biopsy revealed nodal involvement, and the patient underwent wide local excision followed by adjuvant immunotherapy. This report highlights the diagnostic challenges posed by AM, particularly in younger patients and in regions where such cases are rarely reported. Accurate diagnosis requires a high index of suspicion and a multimodal diagnostic approach. Early recognition and intervention are crucial for improving patient outcomes in this aggressive malignancy.

## Introduction

Amelanotic melanoma (AM) is a rare and aggressive type of melanoma that lacks the characteristic pigmentation found in most melanomas, making it extremely challenging to diagnose, leading to delayed intervention and poor outcomes [1]. The histological subtypes of AM include superficial spreading melanoma (SSM), nodular melanoma (NM), lentigo maligna melanoma (LMM), and acral lentiginous melanoma (ALM) [2]. AM accounts for 1-8% of all melanoma cases, while NM represents 22-50% of all AM cases. This makes NM a rare subtype, emphasizing the importance of early identification and treatment, as highlighted in this report [3,4]. Reliance on standard visual examination to detect melanoma may be inadequate, and, accordingly, dermoscopic assessment, histopathology, and immunohistochemical staining have to be relied upon for an accurate diagnosis [5]. Also, given the aggressive behavior of the AM, these tumors tend to be diagnosed at a later stage, with increased Breslow thickness; hence, early detection is key for enhancing prognosis and outcome [6].

In this report, which features the clinical presentation, diagnostic challenges, and treatment of AM, we aim to emphasize the importance of clinical suspicion and multimodal assessment in detecting this subtle yet potentially lethal malignancy, particularly given the limited number of cases reported in the United Arab Emirates (UAE) and the broader Middle East and North Africa (MENA) region. We present a case of NM in a 39-year-old Filipino female who was diagnosed and managed in the UAE.

## Case presentation

A 39-year-old Filipino female presented to the surgical oncology clinic with a gradually enlarging, asymptomatic skin-colored lesion on her right patella. The lesion had been present for approximately four years and had begun enlarging more rapidly over the past year. It had been initially misdiagnosed as a dermatofibroma and treated with three corticosteroid injections, which had failed to cure it. It had also been managed with antibiotics due to a presumptive diagnosis of infection. The patient denied systemic symptoms and reported no family history of skin malignancies or other chronic illnesses. She also had no history of regular sun exposure. No prior trauma was reported to the affected site. She had no known chronic illnesses. She had smoked one pack of cigarettes every three days for 12 years; she had quit four years ago and used alcohol occasionally.

**Figure 1:**
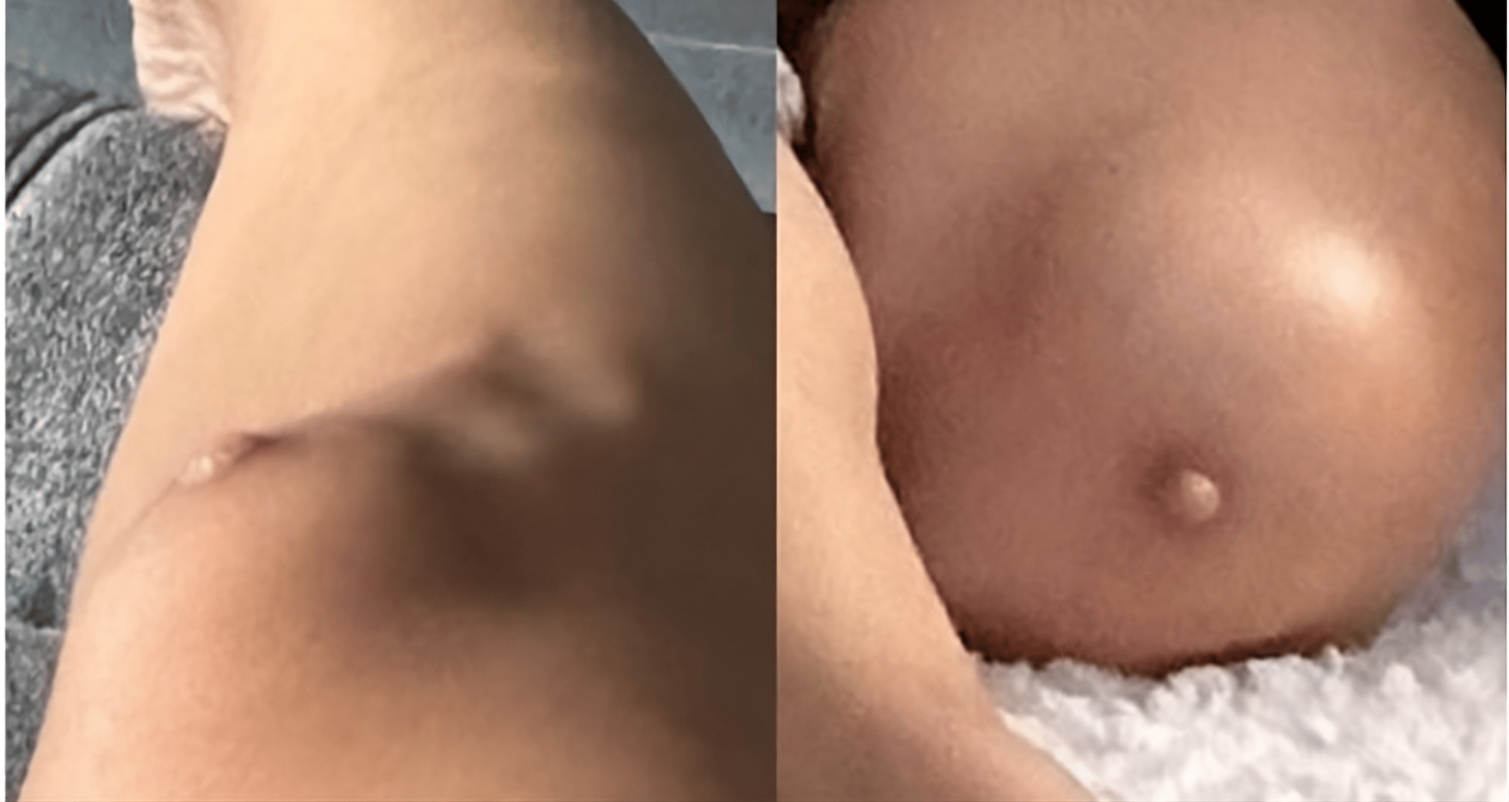
Right patellar region showing a firm, skin-colored dermal lesion

The clinical examination revealed a firm, skin-colored lesion over the right patella that was non-tender and non-ulcerated (Figure 1). There was no palpable regional lymphadenopathy. Systemic examination was unremarkable, and routine preoperative laboratory investigations were within normal limits. Given the suspicion of malignancy, an excisional biopsy was performed for histopathological evaluation.

A 1.5 × 1 × 0.6 cm well-circumscribed, tan-colored dermal lesion was excised. Microscopic examination revealed a nodular dermal tumor composed of fascicles of spindle and epithelioid cells, displaying moderate nuclear irregularity without evident mitotic activity. Histopathology revealed a Breslow's thickness of 5 mm, Clark level IV, and high mitotic rate (4-5 mitoses/mm²), indicating a high-risk T4a stage. Immunohistochemistry was positive for SOX10 and S100, supporting the diagnosis of spindle cell melanoma. Immunohistochemical studies showed that the tumor cells were negative for Melan-A, HMB-45, PRAME, cytokeratin, CD34, and Factor XIIIa. The Ki-67 proliferative index was elevated in the deeper portion of the lesion. There was no evidence of reportable somatic alterations in BRAF (V600E), KIT C.A2447T (Asp816Val), CDKN2A, NF1, KRAS, and NRAS. The final diagnosis was confirmed to be cutaneous amelanotic nodular melanoma of the right knee, spindle/epithelioid type, staged at type IIIb.

**Figure 2:**
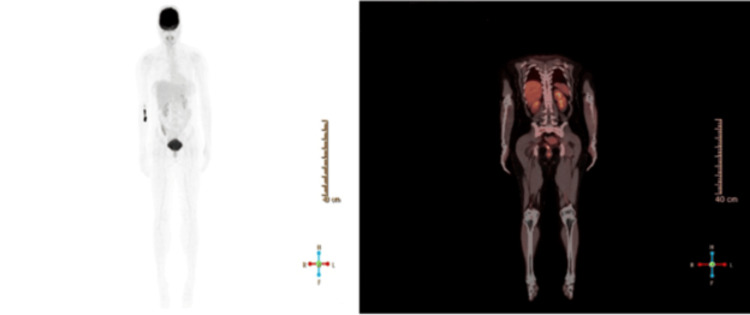
PET imaging demonstrating no signs of metastasis PET: positron emission tomography

Further workup included a PET scan, which showed no FDG-avid lesions or evidence of metastasis in skin, lymph nodes, or visceral organs (Figure 2), and a CT Scan, which revealed incidental, mild hepatomegaly and bilateral basal atelectatic bands and was otherwise unremarkable.

Due to the lesion’s depth, margin involvement, and high-risk histopathology, the multidisciplinary team (MDT) recommended a wide local excision along with sentinel lymph node biopsy.

**Figure 3:**
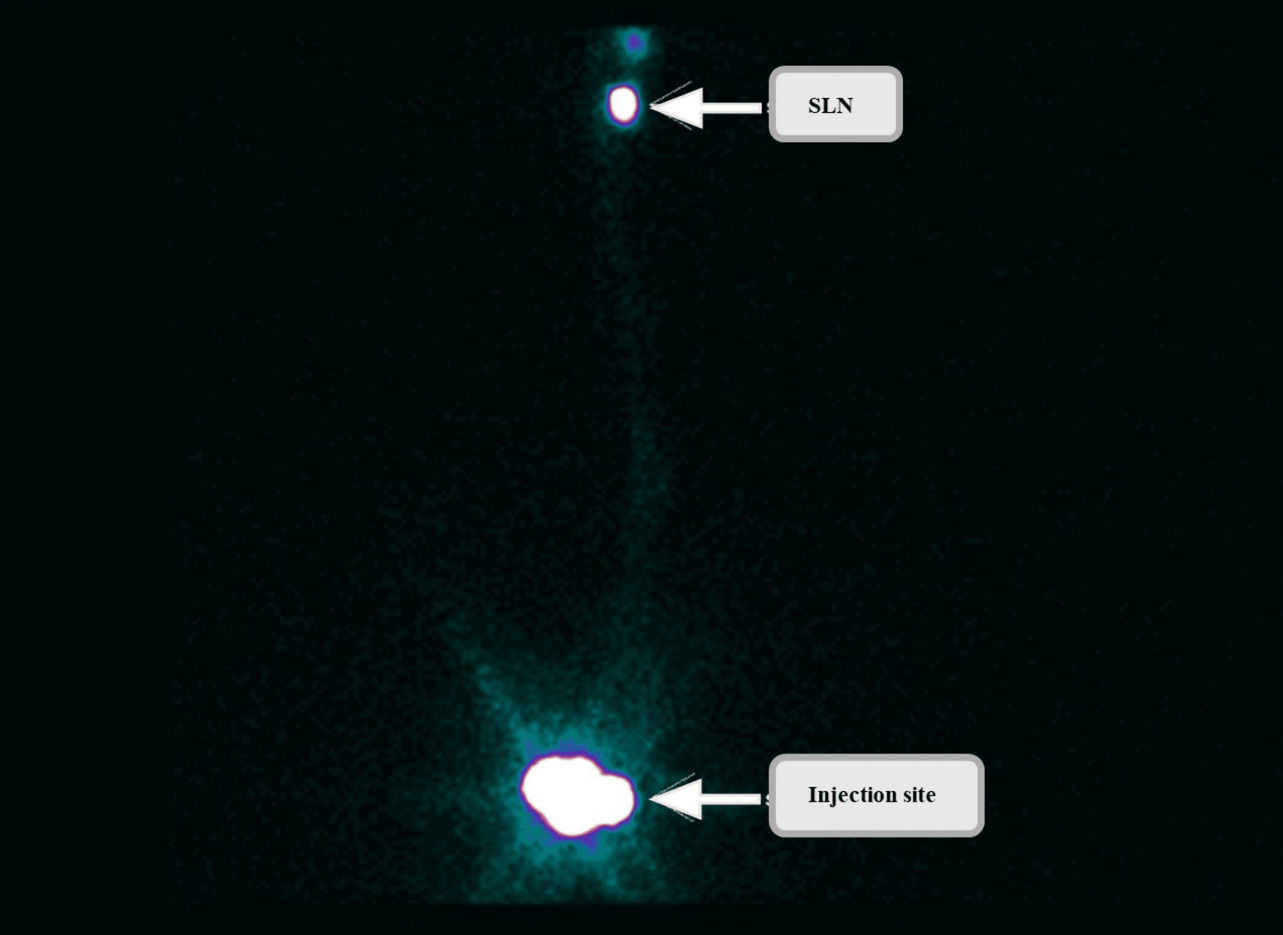
Scintigraphic image following injection of 2.5 mCi of Tc-99m sulfur colloid demonstrating localization of sentinel lymph node

**Figure 4:**
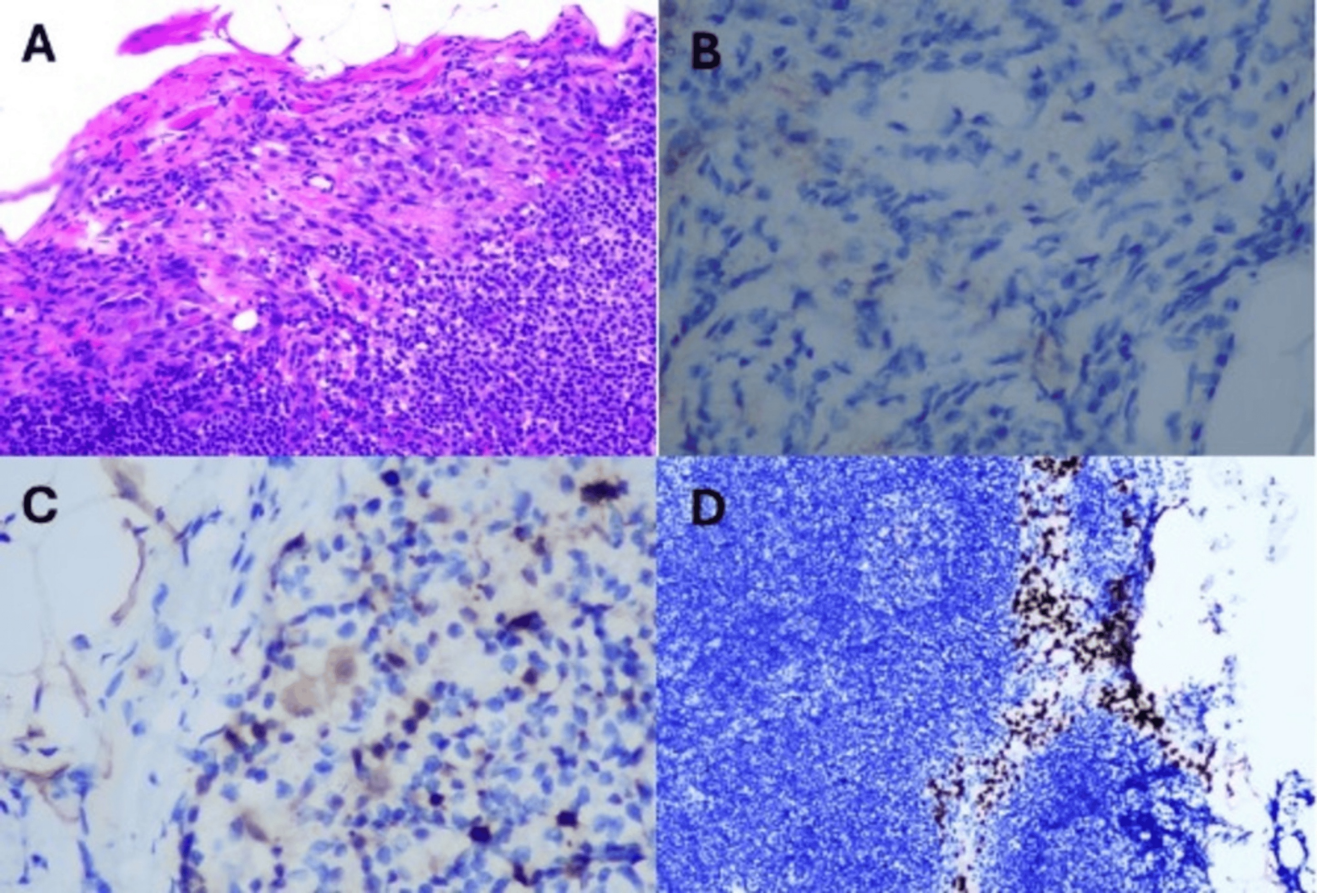
(A) Histological section demonstrating metastatic melanoma cells within the lymph node. (B) Melanin A stain shows negative expression. (C) S100 positive staining. (D) SOX10 positive staining

Preoperative scintigraphy was performed, involving an injection of 2.5 mCi of Tc-99m sulfur colloid, intradermally around the scar site (divided into 4 doses), and the sentinel lymph nodes were marked by nuclear medicine (Figure 3). Intraoperatively, a wide excision of a 1 cm margin circumferentially was made and deepened to 2 cm to the fascia, and a subcutaneous injection of methylene blue was made around the scar. The sentinel lymph nodes were identified using a Gamma probe. Right inguinal lymph node dissection with vascular ligation and excisional biopsy was performed, and the wound was closed using Monocryl sutures and Dermabond. Sentinel lymph node biopsy confirmed deposits in the lymph node. Sentinel lymph node biopsy showed a 2 mm macrometastasis without extracapsular spread, corresponding to stage PN1b (Figure 4).

The patient was further treated with adjuvant pembrolizumab. Notably, she developed grade II immune-related colitis after two cycles, which was managed effectively with corticosteroids. Follow-up calprotectin levels dropped from 299 to 27 μg/g of feces, and treatment was resumed with good tolerance.

## Discussion

Malignant melanoma (MM) remains a significant public health concern, causing high mortality and morbidity worldwide, with the global burden projected to reach 510,000 new cases by 2040 [7]. AM is an aggressive form of MM that accounts for 1-8% of all melanoma cases. Notably, the nodular subtype of AM accounts for 22-50% of all AM cases [3,4]. NM in particular was associated with worse prognosis when compared to other subtypes: ALM, LMM, and SSM [8]. AM is characterized by the lack of pigment and is subdivided into four histologically different variants. The least to most common subtypes of AM are as follows: ALM (1-2%), LMM (5-10%), NM (20%), and SSM (70%) [2]. AM is particularly rare in the MENA region, which has led to limited awareness and diagnostic delays.

Risk factors of AM are similar to those of typical melanoma, which is commonly observed in fair-skinned individuals with frequent sun exposure [9], and the median age at diagnosis is approximately 67 years. Sun exposure and advanced age have been identified as the most important clinical risk factors [10]. In our patient, despite lacking conventional risk factors for melanoma, such as fair skin, advanced age, family history, or significant sun exposure, the lesion was biopsied due to its progression in size. It was subsequently confirmed as NM, a rare and aggressive subtype, making this case a diagnostic challenge. Recent studies have indicated a correlation between alcohol consumption and an increased risk of MM incidence, which aligns with our patient’s history [11].

The ABCDE (Asymmetry, Border irregularity, Color variation, Diameter >6 mm, and Evolution) criteria are widely used to detect conventional melanomas. However, due to AM’s lack of pigment and often subtle clinical features, these criteria are frequently ineffective, leading to delayed diagnosis. Instead, clinicians may rely on dermatoscopy, which enhances visualization of vascular patterns and structureless zones characteristic of AM, and the CUBED (Coloured lesion, Uncertain diagnosis, Bleeding lesion on the foot or under the nail, Enlargement of a lesion, and Delay in healingacronym) acronym as a more effective tool in clinical screening (Table 1) [12].

**Table 1:** CUBED acronym useful for detecting atypical presentation of melanoma* ^*^12 Criteria: If 2 or more of the above features are present in a cutaneous lesion, the risk of potential melanoma is high, and referral to dermatology is warranted

Code	Clinical Indicator
C	Colored skin
U	Uncertain diagnosis
B	Bleeding ulcers in the foot/under the nail
E	Enlargement of the ulcer despite treatment
D	Delay in healing >2 months

AM is often asymptomatic but may manifest variably depending on its histological subtype. The gold standard for diagnosis is a combination of histological examination and immunohistochemistry staining, as histology alone is not sufficient due to the diverse cytological features that AM can present [13]. Histologically, AM typically shows epithelioid, spindle, or desmoplastic cell morphology, characterized by prominent nucleoli, a high mitotic rate, and a lack of pigmentation [2]. In our case, the tumor showed positivity for S100 and SOX10 but was negative for common melanocytic markers such as HMB-45, Melan-A, and PRAME. This immunoprofile underscores the importance of a broad panel of stains to confirm AM diagnosis when typical markers are absent. 

AM is diagnostically challenging due to its nonspecific appearance, often mimicking benign lesions like dermatofibroma, seborrheic keratosis, or pyogenic granuloma. Its lack of classic melanoma features can delay diagnosis and treatment [3]. Multiple high-risk features are associated with poorer outcomes in patients with AM, including advanced age, large tumor size (T4), higher nodal involvement (N-stage), presence of metastasis, ulceration, increased mitotic activity, and greater Breslow thickness [13]. In our case, the lesion demonstrated deep invasion, measuring 5 mm in Breslow thickness, which correlates with a higher risk of metastasis [14]. Another common factor that influences outcomes is nodal involvement, which was evident in our patient. These findings highlight the critical need for early detection, as delays can lead to deeper invasion and metastasis. Prompt recognition and intervention significantly improve prognosis and overall survival in AM patients.

The cornerstone of treatment for AM is surgical excision. According to guidelines published in the Annals of Surgery, melanomas with a thickness greater than 2 mm should be excised with 2 cm margins [15]. As our patient's melanoma measured 5 mm in depth, a 2 cm margin was appropriately used during excision. In our case, the presence of high-risk features warranted the initiation of adjuvant therapy following surgical excision. Per the NCCN Guidelines for Cutaneous Melanoma, our patient received adjuvant systemic therapy following complete surgical resection of stage III AM. This approach aligns with current recommendations aimed at reducing the risk of recurrence and improving disease-free survival in patients with resected stage III melanoma. Unlike neoadjuvant therapy [16], which is administered before surgery to shrink tumors, adjuvant therapy is given postoperatively to reduce the risk of recurrence. Systemic treatments such as immune checkpoint inhibitors have become a cornerstone in managing advanced AM, with agents like nivolumab and pembrolizumab showing great promise in improving survival and disease control [17,18].

## Conclusions

AM remains a diagnostic and therapeutic challenge due to its atypical presentation and lack of pigmentation. This report underscores the need for heightened clinical suspicion, particularly in younger individuals with persistent skin lesions. Accurate diagnosis relies heavily on histopathological assessment combined with an extensive immunohistochemical panel. The presence of high-risk features in our case necessitated surgical excision followed by adjuvant immunotherapy. Hence, early detection, a comprehensive and multidisciplinary approach, and timely management are critical to improving outcomes in patients with this aggressive melanoma subtype.
